# Supplementary material for: Quantification of Visceral Fat at the L5 Vertebral Body Level in Patients with Crohn’s Disease Using T2-Weighted MRI
Source: Bioengineering (Basel). 2024 May 22;11(6):528. doi: 10.3390/bioengineering11060528 (PMC11200797; doi:10.3390/bioengineering11060528)
Supplement: Supplementary file 1 [file bioengineering-11-00528-s001.zip › bioengineering-3016964-supplementary.pdf]

bioengineering

MDPI

Article

Quantification of Visceral Fat at the L5 Vertebral Body Level in Patients with Crohn's Disease Using T2-Weighted MRI

Favour Garuba [1], Aravinda Ganapathy [1], Spencer McKinley [1], Karan H. Jani [2], Adriene Lovato [2], Satish E. Viswanath [3], Scott McHenry [4], Parakkal Deepak [4] and David H. Ballard [2,*]

1 School of Medical Education, Washington University School of Medicine in St. Louis, St. Louis, MO 63110, USA; f.garuba@wustl.edu (F.G.); aganapathy@wustl.edu (A.G.); mckinley.s@wustl.edu (S.M.)
2 Mallinckrodt Institute of Radiology, Washington University School of Medicine in St. Louis, St. Louis, MO 63110, USA; jani@wustl.edu (K.H.J.); lovato@wustl.edu (A.L.)
3 Department of Biomedical Engineering, School of Engineering, Case Western Reserve University, Cleveland, OH 44106, USA; satish.viswanath@case.edu
4 Division of Gastroenterology, Washington University School of Medicine in St. Louis, St. Louis, MO 63110, USA; smchenry@wustl.edu (S.M.); deepak.parakkal@wustl.edu (P.D.)
* Correspondence: davidballard@wustl.edu; Tel.: +1-(314)-362-2928; Fax: +1-(314)-747-4671

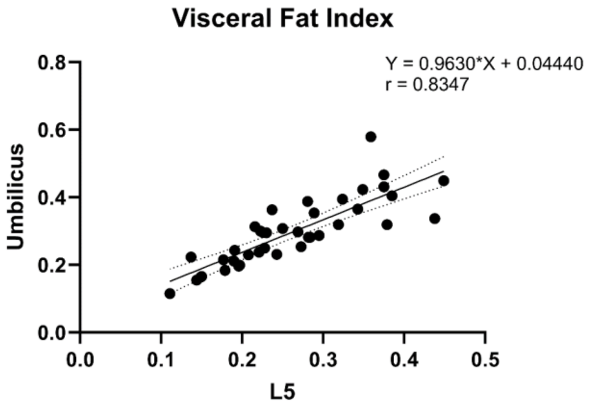

Figure S1. Correlation of Visceral Fat Index Measurements at L5 vs Umbilicus Levels without exclusion of 7 patients.

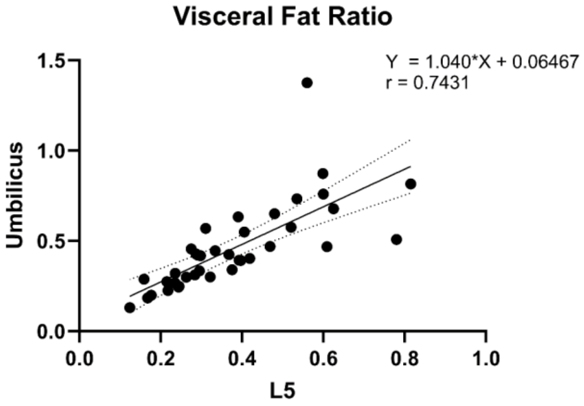

Figure S2. Correlation of Visceral Fat Ratio Measurements at L5 vs Umbilicus Levels without exclusion of 7 patients.
